# EGFR inhibition in non-small cell lung cancer: current evidence and future directions

**DOI:** 10.1186/2050-7771-1-2

**Published:** 2013-01-16

**Authors:** Alexander Chi, Scot Remick, William Tse

**Affiliations:** 1 Department of Radiation Oncology, West Virginia University, Morgantown, WV, 26506, USA; 2 Mary-Babb Randolph Cancer Center, West Virginia University, Morgantown, WV, 26506, USA; 3 Department of Hematology and Oncology, West Virginia University, Morgantown, WV, 26506, USA

**Keywords:** EGFR, NSCLC, EGFR mutations, Radiotherapy, Resistance

## Abstract

EGFR inhibition has emerged to be an important strategy in the treatment of non-small cell lung cancer (NSCLC). Small molecule tyrosine kinase inhibitors (TKIs) and mono-clonal antibodies (mAbs) to the EGFR have been tested in multiple large randomized phase III studies alone or combined with chemotherapy, as well as small phase I-II studies which investigated their efficacy as radiosensitizers when combined with radiotherapy. In this review, we described the current clinical outcome after treatment with EGFR TKIs and mAbs alone or combined with chemotherapy in advanced stage NSCLC, as well as the early findings in feasibility/phase I or II studies regarding to whether EGFR TKI or mAb can be safely and effectively combined with radiotherapy in the treatment of locally advanced NSCLC. Furthermore, we explore the potential predictive biomarkers for response to EGFR TKIs or mAbs in NSCLC patients based on the findings in the current clinical trials; the mechanisms of resistance to EGFR inhibition; and the strategies of augmenting the antitumor activity of the EGFR inhibitors alone or when combined with chemotherapy or radiotherapy.

## Introduction

The epidermal growth factor receptor (EGFR), a 170 kD transmembrane protein consisted of a N-terminus extracellular ligand-binding site, a hydrophobic transmembrane domain, and a C-terminus intracellular region with tyrosine kinase activity, is the first of the ErbB family of receptor tyrosine kinases (RTKs) [1,2]. The other members include ErbB2 (HER2/neu), ErbB3 (HER3), and ErbB4 (HER4). These receptors trigger downstream signaling pathways which lead to multilayered, complex interactions resulting in combinatorial responses. Disruption of these pathways was found to cause malignant transformation [1]. The EGFR is activated through ligand-induced homo or heterodimerization of the receptor with other receptors of the ErbB family under physiologic conditions, but can also be activated due to receptor over-expression, increase of EGFR gene copy number, and activating mutations [3]. EGFR activation has been shown to play a key role in tumor cell proliferation, apoptosis, tumor-induced angiogenesis, metastasis, and DNA damage repair after cytotoxic insults [1,4]. This makes it an attractive target in cancer therapy; and its inhibition a strategy for augmentation of the efficacy of chemotherapy and radiotherapy. Since the initial discovery of the EGFR in 1962, a class of antibodies blocking the EGFR’s extra-cellular ligand binding site to prevent receptor activation, and to down-regulate EGFR expression at the cell surface through antibody mediated receptor dimerization; and low molecular weight tyrosine kinase inhibitors, which competes with ATP to bind to the intra-cellular tyrosine kinase portion of the receptor to abrogate the receptor’s catalytic activity to activate downstream signaling pathways, have been developed [1,4-6]. Among them, reversible small molecule (SM) tyrosine kinase inhibitors (TKIs), Gefitinib and Erlotinib, and the mono-clonal antibody (mAb) against EGFR, Cetuximab, have been the most thoroughly investigated. Both Cetuximab and the SM TKIs have been shown to have significant antitumor activity in various EGFR over-expressing cancers, and to enhance the potency of chemotherapy or radiotherapy in various pre-clinical and early phase I or II clinical studies [1,6-8].

As the leading cause of cancer mortality worldwide, 50-80% of non-small cell lung cancer (NSCLC) is associated with EGFR overexpression; with 65% of them also found to have an increased EGFR gene copy number [9-11]. Given the high percentage of NSCLC being diagnosed in the advanced stage with a poor survival outcome, EGFR inhibition alone or combined with other approaches in the treatment of NSCLC become a very attractive strategy which has been validated in pre-clinical and preliminary clinical trials [12-14]. This led to further phase III randomized studies assessing the efficacy of combining a SM TKI or Cetuximab with chemotherapy as the first line treatment of stage IIIB-IV NSCLC; and some feasibility/phase I & II studies on their combination with radiotherapy, or chemo-radiation in the treatment of local-regional disease. In the following sections, we will describe the current clinical evidence on the utility of TKIs and Cetuximab with chemotherapy in the treatment of advanced stage NSCLC, their role in the treatment of loco-regionally confined NSCLC, such as radiosensitization; patient selection, and potential strategies of further enhancing their efficacy.

## EGFR TKIs alone or combined with chemotherapy as first line treatment

Numerous phase III studies on TKI alone or combined with chemotherapy as first line treatment for stage IIIB-IV NSCLC have been conducted since Gefitinib has been approved by the Food and Drug Administration (FDA) in 2003, and Erlotinib has been approved by the FDA in 2004 for chemotherapy-resistant stage IIIB-IV NSCLC [14-23]. In 2004, two large phase 3 randomized studies evaluating the benefit of adding Gefinitib to chemotherapy as first line treatment for stage IIIB-IV NSCLC have been reported [15,16]. In INTACT 1, 1093 patients were randomized to placebo, Gefitinib 250 mg/day, and Gefitinib 500 mg/day after up to six cycles of Cisplatin and Gemcitabine. No differences in objective response rate (ORR), progression free survival (PFS), or overall survival (OS) was found between the 3 arms after median follow up of 15.9 months. The treatment was very well tolerated with no difference in the incidence of interstitial lung disease (ILD) reported and an overall incidence of ILD <1% [15]. In a parallel study, INTACT 2, similar findings were obtained after randomizing 1037 patients to placebo, Gefitinib 250 mg/day, and Gefitinib 500 mg/day after up to 6 cycles of Carboplatin and Paclitaxel in as first line treatment for stage IIIB/IV patients [16]. The addition of Gefitinib was again very well tolerated with only 0.9%, 2.1%, and 1.5% of ILD found in the three arms, respectively. In the subsequent year, a randomized trial (TRIBUTE) comparing first line Carboplatin and Paclitaxel up to 6 cycles and the same regimen combined with concurrent and maintenance Erlotinib (150 mg/day) in 1079 patients [17]. In this study, no difference in ORR, PFS, or OS was found between the two arms. However, a survival benefit from maintenance Erlotinib was found in patients who lived longer than 4 months, which suggested a benefit from maintenance therapy with Erlotinib. There were 5 cases of ILD-like in the Erlotinib arm (1.0%) and 1 on the chemotherapy arm (0.2%). All cases were fatal. Despite the overall negative findings in this study, never smokers, who tend to be younger, female, and have adenocarcinomas when compared with prior/current smokers, were found to have a median survival of 22.5 months with Erlotinib treatment and only 10.1 months when treated with placebo.

Despite the disappointment with the INTACT and TRIBUT studies, the importance of selecting patients who will respond to TKIs has been recognized, which is suggested by the prolonged survival observed in never smokers [16,17]. This concept is also supported by the findings of in-frame deletions in exon 19 and missense mutations in exon 21 of the EGFR gene which lead to increased signaling through the EGFR pathway, and increased drug affinity at the EGFR’s intra-cellular tyrosine kinase ATP-binding site [24,25]. Furthermore, the above mentioned EGFR mutations are more often found in never-smokers as well [17].

To further classify the patients who will respond to EGFR TKIs, a phase III randomized study (IPASS) comparing upfront Geftinib and Carboplatin/Paclitaxel in stage IIIB-IV patients with the following characteristics has been conducted in East Asia: never or former light smokers with a diagnosis of adenocarcinoma [18]. A total of 1217 patients were randomized in this study. After a median follow up of 5.6 months, a superior 1 year PFS with Gefitinib was found (24.9% *vs*. 6.7%, *p* <*0*.*001*). Better ORR associated with Gefitinib was also reported (43% *vs*. 32.2%, *p* <*0*.*001*). Among 437 patients whose EGFR mutation data can be evaluated, 261 had EGFR mutations identified. Out of these 261 patients, 53.6% had exon 19 deletions, and 42.5% had a missense mutation at exon 21. A predictor of TKI resistance [26,27], a mutation at exon 20 (T790M) was also identified in 4.2% of these patients. Among patients who harbored activating EGFR mutations on exon 19 and 21, a noticeably increased ORR in patients who received Gefitinib was found when compared with those who received chemotherapy (71.2% *vs*. 47.3%, *p* <*0*.*001*). The opposite was found in patients without EGFR mutations (1.1% *vs*. 23.5%, *p* <*0*.*001*). No significant difference in toxicity profile between the two treatment arms was found. This study established that EGFR mutation, such as those on exon 19 and 21, can be used as a predictive marker for response to EGFR TKIs, which may be more effective than chemotherapy in patients with these mutations. Its findings were further validated in several other phase 3 randomized studies [19-22]. All these studies included patients with sensitive EGFR mutations in exons 19 and 21 only. All four studies confirmed the superior response and PFS associated with an EGFR TKI over chemotherapy in patients with activating EGFR mutations. The details of these studies along with the studies described above are shown in Table 1. One more recent report of a trial (TORCH) comparing first line Erlotinib and Cisplatin/Gemcitabine (CG) followed by either CG or Erlotinib in unselected patients with stage IIIB-IV patients has demonstrated negative outcomes associated with Erlotinib after a median follow up of 24.3 months [23]. Thus, further validating the need to select patients based on specific EGFR mutations in NSCLC patients that are associated with drug sensitivity to EGFR TKIs.

**Table 1:** **Phase III Studies Investigating the efficacy of EGFR inhibition alone or as part of the 1**^**st**^**line treatment for stage IIIB-IV NSCLC**

**Study**	**Stage**	**Study Arms**	**# Pts**	**ORR**	**Median PFS**	**Median OS**
***TKI***						
INTACT 1 [15]	IIIB-IV	Cisplatin + Gemcitabine (CG) vs. CG + Gefitinib 250 mg/d vs. CG + Gefitinib 500 mg/d	363 *vs*. 365 *vs*. 365	47.20% *vs*. 51.20% *vs*. 50.30%, *p* = *ns*	6.0 mo *vs*. 5.8 mo *vs*. 5.5 mo, *p* = *0*.*7633*	10.9 mo *vs*. 9.9 mo *vs*. 9.9 mo, *p* = *0*.*4560*
INTACT 2 [16]	III-IV	Carboplatin + Paclitaxel (CP) *vs*. CP + Gefitinib 250 mg/d *vs*. CP + Gefitinib 500 mg/d	345 *vs*. 345 *vs*. 347	28.70% *vs*. 30.40% *vs*. 30.00%, *p* = *ns*	5.0 mo *vs*. 5.3 mo *vs*. 4.6 mo, *p* = *0*.*0562*	9.9 mo *vs*. 9.8 mo *vs*. 8.7 mo, *p* = *0*.*6385*
TRIBUTE [17]	IIIB-IV	Carboplatin + Paclitaxel (CP) vs. CP + Erlotinib; followed by Erlotinib maintenance	540 *vs*. 539	19.30% *vs*. 21.50%, *p* = *0*.*36*	4.9 mo *vs*. 5.1 mo, *p* = *0*.*36*	10.5 mo *vs*. 10.6 mo, *p* = *0*.*95*
IPASS [18]*Nonsmokers or former light smokers with adenocarcinoma*	IIIB-IV	Carboplatin + Paclitaxel *vs*. Gefitinib	608 *vs*. 609	32.20% vs. 43.00%, *p* <*0*.*001*	6.7% vs. 24.9% at 1 year, *p* <*0*.*001*	17.3 mo vs. 18.6 mo
WJTOG3405 [19]*Sensitive EGFR mutation* + *only*	IIIB-IV, postoperative recurrent	Gefitinib *vs*. Cisplatin + Docetaxel	86 *vs*. 86	62.10% *vs*. 32.20%, *p* <*0*.*0001*	9.2 mo vs. 6.3 mo, *p* <*0*.*0001*	30.9 mo *vs*. not reached, *p* = *0*.*211*
OPTIMAL [20]*Sensitive EGFR mutation* + *only*	IIIB-IV	Erlotinib *vs*. Carboplatin + Gemcitabine	82 *vs*. 72	83% *vs*. 36%, *p* <0.0001	13.1 mo vs. 4.6 mo, *p* <*0*.*0001*	
NEJ002 [21]*Sensitive EGFR mutation* + *only*	IIIB-IV, postoperative recurrent	Gefitinib vs. Carboplatin/ Paclitaxel	114 *vs*. 114	73.7% *vs*. 30.7%, *p* <*0*.*001*	10.8 mo *vs*. 5.4 mo, *p* <*0*.*001*	27.7 mo *vs*. 26.6 mo, *p* = *0*.*483*
EURTAC [22]*Sensitive EGFR mutation* + *only*	IIIB-IV	*Erlotinib v*s. Platinum based chemotherapy	86 *vs*. 87	63% *vs*. 18%, *p* <0.001	9.7 mo *vs*. 5.2 mo, *p* <*0*.*0001*	19.3 mo vs. 19.5 mo, *p* = *0*.*87*
TORCH [23]	IIIB-IV	Erlotinib vs. Cisplatin/ Gemcitabine as 1^st^ line treatment, and the opposite as 2^nd^ line therapy	373 *vs*. 371	20.3% vs. 32.6%; 2^nd^ line Erlotinib vs. 2^nd^ line chemo: 4.7% vs. 10.5%; *p* <*0*.*001*	6.4 mo *vs*. 8.9 mo	8.7 mo *vs*. 11.6 mo
***Cetuximab***						
FLEX [33]	IIIB-IV	Cisplatin + Vinorelbine (CV) *vs*. CV + Cetuximab	568 *vs*. 557	29% *vs*. 35%, *p* = *0*.*010*	4.8 mo *vs*. 4.8 mo, *p* = *0*.*39*	10.1 mo *vs*. 11.3 mo, *p* = *0*.*044*
BMS099 [34]	IIIB-IV	Carboplatin + Taxane (CT) *vs*. CT + Cetuximab	338 *vs*. 338	17.20% *vs*. 25.70%, *p* = *0*.*007*	4.24 mo *vs*. 4.40 mo, *p* = *0*.*2358*	8.38 mo *vs*. 9.69 mo, *p* = *0*.*169*

*Abbreviations*: ORR: objective response rate; PFS: progression free survival; OS: overall survival.

## EGFR TKIs as 2^nd^ line or maintenance therapy

While effective only in EGFR mutation positive patients as first line treatment, both Gefitinib and Erlotinib have been shown to achieve similar ORR, PFS, and OS when compared with 2^nd^ line chemotherapy in randomized phase 3 studies [28,29]. EGFR FISH-negative tumors were found to have a higher risk of disease progression after Erlotinib in one study [29]. When compared with placebo as maintenance therapy, Erlotinib treatment led to increased PFS, and EGFR expression was significantly correlated with PFS [30]. In addition, EGFR mutation s were associated a large improvement in PFS with the addition of Erlotinib as maintenance therapy.

## Cetuximab combined chemotherapy as first line treatment

While a large amount of clinical evidence on how to most effectively incorporate EGFR TKIs into the management of advanced NSCLC has been generated, the clinical experience with mono-clonal antibodies in advanced NSCLC is just emerging in recent years. Cetuximab as a single agent generated minimal response in advanced NSCLC [31]. However, its addition to first line Cisplatin and Vinorelbine was shown to increase tumor response in stage IIIB-IV NSCLC when compared with the same chemotherapy alone in a phase II study [32]. Two randomized phase III studies on the combination of Cetuximab with chemotherapy as first line treatment have been reported subsequently [33,34]. In the FLEX study, which randomized 1125 patients with stage IIIB-IV NSCLC to Cetuximab delivered concurrently with up to 6 cycles of Cisplatin and Vinorelbine and until disease progression and the same chemotherapy alone, the addition of Cetuximab led to significantly increased ORR (35% *vs*. 29%, *p* = *0*.*010*) and median survival (11.3 *vs*. 10.1 months, *p* = *0*.*044*). Only 10% of the patients experienced an acne-like rash on the Cetuximab arm, and no >grade 3 acne-like rash was observed in this study. The superior response associated with the addition of Cetuximab to first line chemotherapy was confirmed in another phase III randomized study (BMS 099) randomizing 676 patients to Carboplatin, plus a taxane or Caboplatin-based chemotherapy combined with Cetuximab, which is given till disease progression. However, no survival benefit was demonstrated in that study (Table 1). Only 10.5% grade 3 or 4 acne-like rash was observed in this study.

## Patient selection for EGFR TKIs or mAb in NSCLC: the current evidence

Given the studies summarized above, it is clear that patient selection for EGFR TKI or mAb is essential as they are only effective in a group of patients with certain biological traits. The association between EGFR mutations and the efficacy of SM TKIs in phase 3 randomized studies is direct evidence for this. As a result, predictive biomarkers for efficacy of TKI or mAb treatment have been sought in patients from various phase II/III and prospective studies (Table 2). Among studies of a TKI alone as 1^st^, 2^nd^, 3^rd^ line treatment or maintenance therapy, EGFR mutation was identified most frequently and consistently as a predictor of tumor response, and survival outcome [35-40]. However, KRAS mutation, pAKT and HER-2 expression were also found to be possible predictive biomarkers for survival outcome following TKI treatment in selected studies [36-38]. When used in combination with chemotherapy, EGFR mutation and KRAS mutation and EGFR gene copy number are identified to be potential predictive biomarkers for TKI efficacy [41,42]. It is also found that the EGFR gene copy number predictive value depended on the EGFR mutation status in the IPASS trial (Table 2).

**Table 2:** Predictive biomarkers in the major clinical trials

**Study**	**Study Arms**	**Predictive Biomarkers**
***TKI***		
INTEREST [35]	Gefitinib vs. Docetaxel in previous treated patients	EGFR mutation predicted for longer PFS and higher ORR (*p* <*0*.*05*) and high EGFR copy number predicted for higher ORR (*p* <0.05) with Gefinitib *vs*. Docetaxel. KRAS mutation was not predictive for response or survival.
SATURN [36]	Maintenance Erlotinib vs. observation after 1^st^ line chemotherapy	EGFR IHC, EGFR FISH, KRAS mutation and EGFR CA-SSR1 repeat length status did not predict for drug response. EGFR mutation predicted for PFS, and OS benefit after Erlotinib treatment KRAS mutation predicted for poor PFS.
Italian Phase II study [37]	EGFR-TKI as 2^nd^ line treatment	pAKT and HER-2 expression are the only independent predictors of PFS and OS
ERMETIC [38]	EGFR TKI as 1^st^, 2^nd^, or 3^rd^ line treatment	Median PFS: EGFR mutant 8.4 mo, EGFR wild type 2.3 mo, KRAS mutant 1.9 mo, *p* = *0*.*001*. Median OS: EGFR mutant 14.4 mo, EGFR wild type 5.3 mo, KRAS mutant 4.1 mo, *p* = *0*.*004*.
PUMC prospective study [39]	Gefitinib after failing 1^st^ line chemotherapy	EGFR mutation is the only independent predictor of tumor response.
IDEAL & INTACT trials [40]	IDEAL trials: phase II studies on Gefitinib as 2^nd^ line treatment; INTACT trials: phase III studies investigating the benefit of adding Gefitinib to chemotherapy as 1^st^ line treatment	EGFR mutation predicted for increased response to Gefitinib in IDEAL trials, but not in the INTACT trials. KRAS mutation, PTEN mutation or expression and p53 expression not associated with clinical response to Gefitinib in the IDEAL trials.
TRIBUTE [41]	Carboplatin + Taxol (CT) vs. CT + Erlotinib; followed by Erlotinib maintenance	EGFR mutations (13%) in EGFR exons 18 through 21 were predictive of OS overall; and superior response to CT + Erlotinib (p <0.01). KRAS mutations (21%) at KRAS exon 2 was associated with decreased TTP and OS in the CT + Erlotinib arm.
IPASS (Fukuoka JCO 11) [42]*Nonsmokers or former light smokers with adenocarcinoma*	Carboplatin + Taxol *vs*. Gefitinib	High EGFR gene copy number was associated with increased PFS and ORR after Gefitinib vs. Carbo/Taxol. However, it predicted for poorer PFS in the absence of EGFR mutation. EGFR mutation at Exon 19 and 21 predicted for superior PFS and ORR after Gefitinib.
***Cetuximab***		
SWOG 0342 [43]	Carbo/Taxol + Cetuximab *vs*. Carbo/Taxol; Both followed by Cetuximab for 1 year	Increased EGFR gene copy numbers is associated with superior PFS and OS (*p* <0.05)
BMS099 [44]	Carboplatin + Taxane (CT) *vs*. CT + Cetuximab	EGFR FISH, EGFR IHC, KRAS mutations and EGFR mutations did not predict for ORR, PFS, or OS benefit with the addition of Cetuximab
FLEX [45]	Cisplatin + Vinorelbine (CV) *vs*. CV + Cetuximab	KRAS (19%), EGFR FISH + (37%), PTEN negativity (35%) were of no predictive value. EGFR mutation (15%) predicted for improved OS in both arms (*p* <*0*.*05*).

Among phase II-III studies evaluating the efficacy of Cetuximab, only the EGFR gene copy number and EGFR mutation were found to be potential biomarkers for this mAb’s efficacy (Table 2). Among the two large phase 3 randomized studies (BMS099 & FLEX trials), the EGFR mutation was the only biomarker found to be predictive of superior survival after either chemotherapy combined with Cetuximab or chemotherapy alone in the FLEX trial [44,45].

Based on the evidence generated from the studies described thus far, the EGFR mutation status appear to be the most consistently found predictive biomarker for EGFR TKI or mAb efficacy in advanced NSCLC while other potential markers, such as KRAS mutation and EGFR gene copy number, need to be further evaluated in larger cohort of patients in the future.

## Potential role of EGFR TKI and mAb as radiosensitizers

The EGFR has been known to play a key role in the activation of DNA repair and anti-apoptotic proteins, as well as tumor cell repopulation after irradiation [8,46]. Upon irradiation, increased levels of EGFR auto-phosphorylation has been observed, leading to the activation of the EGFR and its downstream signaling pathways [8]. Furthermore, its expression has been shown to be correlated with radio-resistance and poor outcome after radiotherapy in pre-clinical and clinical studies [8,47]. Thus, adding an EGFR TKI or mAb to inhibit EGFR activation induced by irradiation become a very sound strategy in enhancing the radiocurability of EGFR over-expressing cancers. This has been successfully validated in the treatment of locally advanced squamous cell carcinoma of the head and neck. In a randomized phase 3 study comparing concurrent Cetuximab plus radiotherapy and radiotherapy alone, a 9.2% 5 year overall survival benefit (45.6% vs. 36.4%, *p* <*0*.*05*) was observed with the addition of cetuximab [48]. As an EGFR over-expression cancer, locally advanced NSCLC has been treated with concurrent chemo-radiation with a 5-year survival of approximately 15%, and 5 year loco-regional control of approximately 70% based on major randomized studies [49]. Despite the poor outcome observed, concurrent chemoradiation is often associated with significant toxicity, which prevents further radiation dose escalation. Therefore, combining an EGFR TKI or mAb with radiotherapy or chemoradiation can potentially be a more effective strategy in the treatment of locally advanced NSCLC.

In pre-clinical studies of NSCLC, radiosensitizing effects of both EGFR TKIs and mAbs have been reported [13,50-54]. This has led to a series of studies combining TKIs and mAbs to the EGFR with radiotherapy or chemoradiation. Combining a TKI with radiotherapy or chemoradiation has been shown to be feasible in several studies [55-63]. As shown in Table 3, median survival of >20 months was achieved in several studies [58,59,62,63]. As shown by Wang *et al*., a concurrent TKI and radiotherapy to 70 Gy in stage III-IV patients achieved a median survival of 21.8 months, which is at least comparable to what has been reported after concurrent chemoradiation [58,64,65]. However, the addition of chemotherapy to TKI and radiotherapy may possibly increase the risk of fatal pneumonitis and hematologic toxicities [57,59]. Furthermore, the use of concurrent chemotherapy, radiotherapy and EGFR TKI may not be better than combined TKI and radiotherapy [61]. Disappointing survival outcome is again demonstrated in another phase II study investigating induction chemotherapy followed by concurrent chemotherapy, TKI, and radiotherapy to 74 Gy [60]. This implies the potential risk of increased toxicity when combining chemotherapy, EGFR TKI and radiotherapy; and also the importance of drug treatment sequencing for chemotherapy and TKI. As shown in a different study, which delivers 1 dose of chemotherapy followed by subsequent doses of TKI each week with radiotherapy, median survival appears to be noticeably improved when treatment was delivered in this fashion [63]. Due to the size of the studies, predictive biomarkers have not been consistently identified. However, long term survival and excellent overall survival have been reported in patients who harbor EGFR mutations (deletion on exon 19) and who responded to upfront TKI [55,62]. Again, suggesting the importance of patient selection in the application of TKI’s as radiosensitizers.

**Table 3:** EGFR tyrosine kinase inhibitors or mono-clonal antibodies combined with radiotherapy

**Study**	**Study scheme**	**Stage**	**#Pts**	**RT Dose (Gy)**	**ORR**	**Median PFS**	**Median OS**
**TKI**							
Okamoto *et al *[55]	Gefitinib x 14 days, then given concurrently with RT	III	9	60 Gy	4 pts who completed with PR		2 pts with EGFR mutations lived >5 yrs.
Choong *et al *[56]	Erlotinib + Cisplatin + Etoposide + RT then docetaxel x 3 cycles vs. Carboplatin + Paclitaxel , then Erlotinib + Carboplatin + Paclitaxel + RT	III	17 *vs*. 17	66 Gy	65% *vs*. 59%, *p* = *ns*	13% *vs*. 15% at 3 yrs, *p* = *0*.*9168*	11 mo *vs*. 15 mo, *p* = *0*.*8979*
Rothschild *et al *[57]	Erlotinib + RT or Erlotinib + Cisplatin + RT	III	14	63 Gy	21.4%	6.0 mo	12.7 mo
Wang *et al *[58]	Gefitinib or Erlotinib + RT	III/IV	26	70 Gy	96%	10.2 mo	21.8 mo
Center *et al *[59]	Gefitinib + Docetaxel + RT	III	16	70 Gy		7.1 mo	21.0 mo
Stinchcombe *et al *[60]	Carboplatin + Irinotecan + Taxol followed by Carboplatin/Taxol + Gefitinib + RT	III	23	74 Gy	24%	9 mo	16 mo
Ready *et al *[61]	Carboplatin/Taxol x 2 cycles ± Gefitinib then Gefinitib + RT vs. Carboplatin/Taxol + Gefitinib + RT; both groups are given Gefitinib after RT if w/o severe radiation toxicity.	III	21 *vs*. 39	66 Gy	52.40% vs. 81.60%, *p* = *0*.*034*	13.4 mo vs. 9.2 mo	19.0 mo vs. 13.0 mo
Chang *et al *[62]	Upfront TKI, followed by TKI + multitarget IMRT with helical tomotherapy	IIIB-IV	25	40-50 Gy in 16–20 daily fractions.	84%	16 mo	Not reached, 3 yr OS 62.5%
Komaki *et al *[63]	Carboplatin + Paclitaxel on Monday, followed by Erlotinib for the rest of the week combined with RT; Consolidative Carboplatin + Paclitaxel x 2 cycles were then given	III	46	63 Gy/ 35 daily fractions	80%	14.5 mo	34.1 mo
**Cetuximab *or *** **Nimotuzumab**							
Hughes *et al*[66]	Platinum based chemotherapy followed by Cetuximab + RT	III-IV	12	64 Gy	70%		
Choi *et al *[67]	Weekly Nimotuzumab + RT, then q2 weeks till progression	IIB-IV	15	30 or 36 Gy in 3 Gy fractions	46.70%	5.4 mo	9.8 mo
Bebb *et al *[68]	Weekly Nimotuzumab + RT, then q2 weeks till progression	III-IV	18	30 or 36 Gy in 3 Gy fractions	66%	4 mo	15 mo
Govindan *et al *[69]	Carboplatin + Pemetrexed + RT vs. Carboplatin + Pemetrexed + Cetuximab + RT	III	48 *vs*. 53	70 Gy	77% *vs*. 72%, *p* = *ns*	12.6 mo *vs*. 12.3 mo, *p* = *ns*	21.2 mo *vs*. 25.2 mo, *p* = *ns*
Jensen *et al *[70]	Cetuximab + RT followed by maintemance Cetuximab	III	30	66 Gy		8.5 mo	19.6 mo
Hallqvist *et al *[71]	Cisplatin + Docetaxel followed by Cetuximab + RT	III	75	68 Gy	23.5%		17 mo
Blumenschein *et al *[72]	Carboplatin + Taxol + Cetuximab + RT followed Carboplatin + Taxol + Cetuximab	III	87	63 Gy	62%	12 mo	22.7 mo

A limited number of studies investigating the radiosensitizing effects of a mAb have been reported as well (Table 3). Among them, several have translated into median survival rates comparable to what has observed after concurrent chemoradiation [64,65,69-72]. The addition of Cetuximab or Nimotuzumab to radiotherapy or chemoradiation appear to be well tolerated with >grade 3 pneumonitis reported only in cases which had excessive amount of radiation dose to the normal lung tissue [66-72]. The results from RTOG 0617, a confirmatory intergroup trial evaluating the addition of cetuximab to chemoradiation in stage III NSCLC are eagerly anticipated.

## Future directions

The findings from large phase III randomized studies demonstrated that patient selection is essential in the application of EGFR TKI in the treatment of stage IIIB-IV NSCLC. Patients who harbor a activating EGFR mutation usually responds very well to treatments including EGFR TKI or mAb alone or combined with chemotherapy and/or radiotherapy [35,36,38-42,45,55]. This further confirmed previous findings in phase II studies, which demonstrated higher response rates to EGFR TKIs in patients with activating EGFR mutations than that found after platinum-based chemotherapy, but very poor response in patients with only wild type EGFR expression [73]. The response associated with EGFR mutations is thought to be due mainly to an “addictive” dependence of tumor cells on the EGFR signaling pathway for survival [73]. However, response to the EGFR TKIs may be short lived due possibly to a secondary mutation in the EGFR (T790M), or increased signaling through pathways downstream to the EGFR without its activation [26,27,74]. T790M may increase the GTP affinity in the tyrosine kinase domain of the EGFR, or hindrance to TKI binding to the tyrosine kinase domain [75,76]. As shown by Su *et al*., T790M appears to be more prevalent in patients who has not had any TKI treatment (25.2% - 31.5%), and it becomes even more prevalent after TKI treatment (83.3%) [76]. This finding suggests that TKI treatment selects for NSCLC cells with T790M mutation, and new strategy needs to be developed to overcome T790M related resistance. Further shown by Su *et al*., NSCLC patients who harbor mutations in exons 19, 20, and 21 can still benefit from TKI, with superior PFS to that in patients without any of these mutations [76]. Therefore, TKI is still indicated in patients with activating EGFR mutations on exons 19, and 21 with or without T790M identified while additional treatment should be added to overcome resistance due to T790M. Resistance due to T790M can potentially overcome by second generation irreversible EGFR TKIs, which appear to be well tolerated in early clinical studies [5]. Although did not demonstrated an overall survival benefit, Afatinib, an irreversible EGFR, Her-2, and ErbB4 blocker has led to significant PFS in patients who progressed after a 12 week course of Gefitinib or Erlotinib when compared to placebo in a phase 2b/3 trial [77]. This agent has been shown to be highly effective in adenocarcinoma harboring activating mutations on exon 19 and 21 when compared with Cisplatin and Pemetrexed (CP) approximately doubling the response rate and PFS achieved with CP, which makes first line treatment of choice in this cohort of patients [78].

Increased signaling through the PI3K pathway through MET-trigger ErbB3 signaling in TKI-resistant NSCLC cells overexpressing MET oncogene has been shown to be a classic example of acquired resistance through bypassing the EGFR, which can be overcome through dual EGFR and MET inhibition [74]. This strategy has been shown to be effective in pre-clinical studies. As shown by Nakagawa *et al*., WZ4002, a mutant EGFR-TKI, and E7050, a mutant selective dual inhibitor of Met and VEGFR-2, were able to inhibit tumor growth in Erlotinib resistant NSCLC cells *in vitro* and *in vivo* when given together with successful inhibition of the EGFR, Met, and their downstream PI3K-AKT pathway [79]. The combination of 2^nd^ generation TKIs and MET inhibitors with radiotherapy and/or chemotherapy still need to be further investigated in future studies. In addition to MET signaling stimulated PI3K signaling, BRAF mutation in the RAS-RAF-MEK-MAPK pathway, and loss of PTEN expression may also lead to acquired resistance to an EGFR TKI [80,81].

As a another potential predictive biomarker for a lack of response to EGFR TKIs in NSCLC, KRAS mutation was only shown to be associated with treatment outcome in selected studies [38,41]. It is observed in approximately 30% of adenocarcinomas [82]. Its mutated form often leads to a gain of function in its downstream signaling pathway, in particular, the Raf-MEK-ERK pathway. This subsequently increases MAPK signaling and tumor cell proliferation. In addition, the mutated KRAS also leads to increased TGF-α expression, which further stimulates the EGFR [82]. Although not consistently demonstrated in major randomized phase III studies, KRAS mutations was found to be significantly associated with an absence of response to TKIs in NSCLC in a meta-analysis of 17 studies [83]. The role of KRAS mutation in major studies evaluating the efficacy of Cetuximab has not been clearly shown. Thus, the role of KRAS mutation as a predictive biomarker for poor response to EGFR inhibition needs to be further elucidated.

Due to differences of the EGFR inhibition mechanism by the mAb, which can generate antibody dependent cellular cytotoxicity; and TKIs ability to inhibit multiple types of ErbB receptors, the role of dual blockade should be explored further in the clinical as they have been shown to increase the potency of EGFR inhibition *in vitro* and *in vivo *[1,84]. Targeting downstream signaling pathways to the EGFR directly can also be a potential approach of augmenting the antitumor activity of radiotherapy and chemotherapy with EGFR inhibitors [85,86]. At last, simultaneously blocking the EGFR, its downstream signaling pathway, such as the PI3K pathway, and parallel signaling pathways alone, or combined with chemotherapy and radiotherapy, may lead to maximal antitumor activity and warrants further investigation in the future.

## Competing interests

The authors declare that they have no competing interests.

## Authors’ contributions

AC, SR and WT have generated the idea of conducting this review and AC has writing the manuscript with SR and WT reviewed, given suggestions on modifications, and approved the final version. All authors read and approved the final manuscript.
